# Endocrine Regulation of Compensatory Growth in Fish

**DOI:** 10.3389/fendo.2013.00074

**Published:** 2013-07-01

**Authors:** Eugene T. Won, Russell J. Borski

**Affiliations:** ^1^Department of Biology, North Carolina State University, Raleigh, NC, USA

**Keywords:** compensatory growth, fish, aquaculture, growth hormone, ghrelin, NPY, leptin, insulin-like growth factor

## Abstract

Compensatory growth (CG) is a period of accelerated growth that occurs following the alleviation of growth-stunting conditions during which an organism can make up for lost growth opportunity and potentially catch up in size with non-stunted cohorts. Fish show a particularly robust capacity for the response and have been the focus of numerous studies that demonstrate their ability to compensate for periods of fasting once food is made available again. CG is characterized by an elevated growth rate resulting from enhanced feed intake, mitogen production, and feed conversion efficiency. Because little is known about the underlying mechanisms that drive the response, this review describes the sequential endocrine adaptations that lead to CG; namely during the precedent catabolic phase (fasting) that taps endogenous energy reserves, and the following hyperanabolic phase (refeeding) when accelerated growth occurs. In order to elicit a CG response, endogenous energy reserves must first be moderately depleted, which alters endocrine profiles that enhance appetite and growth potential. During this catabolic phase, elevated ghrelin and growth hormone (GH) production increase appetite and protein-sparing lipolysis, while insulin-like growth factors (IGFs) are suppressed, primarily due to hepatic GH resistance. During refeeding, temporal hyperphagia provides an influx of energy and metabolic substrates that are then allocated to somatic growth by resumed IGF signaling. Under the right conditions, refeeding results in hyperanabolism and a steepened growth trajectory relative to constantly fed controls. The response wanes as energy reserves are re-accumulated and homeostasis is restored. We ascribe possible roles for select appetite and growth-regulatory hormones in the context of the prerequisite of these catabolic and hyperanabolic phases of the CG response in teleosts, with emphasis on GH, IGFs, cortisol, somatostatin, neuropeptide Y, ghrelin, and leptin.

## Introduction: Compensatory Growth Overview

**Figure 1:**
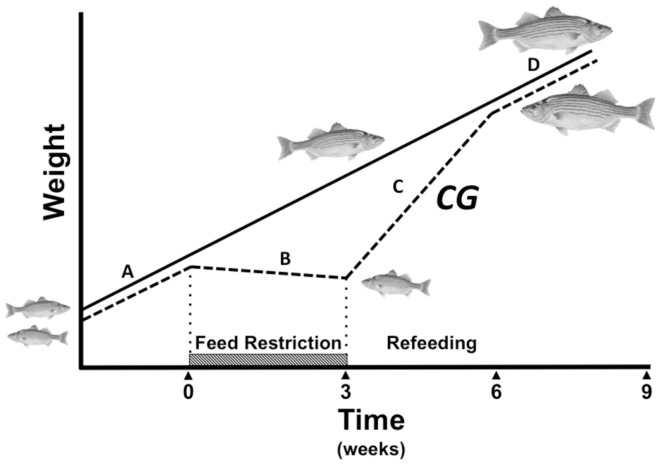
**Compensatory growth (CG) paradigm during fasting and refeeding (dashed line) compared to constant growth rate in fed controls (solid line)**. Normal growth **(A)** is disrupted by feed restriction (hatched bar), which results in a decline in the growth trajectory **(B)** and a size disparity compared to control animals fed a constant regimen. When feeding resumes, hyperphagia and enhanced growth axis activity drive a hyperanabolic phase **(C)** marked by a steeper growth curve than that of constantly fed animals. The CG response potentially allows stunted animals to fully compensate for lost growth opportunity and re-converge in size with constantly fed controls before the growth rate returns to normal **(D)**.

Compensatory growth (CG) is a period of accelerated somatic growth following the alleviation of growth-stunting conditions, that temporarily induces a steeper growth trajectory than that of cohorts not previously exposed to adverse conditions (Figure 1). This phenomenon was seminally documented nearly a century ago (Osborne and Mendel, 1916), and the term “CG” coined 40 years later (Bohman, 1955). CG has been documented in all vertebrate classes; humans (Prader et al., 1963; Boersma and Wit, 1997; Sapolsky, 1998), other mammals (Bohman, 1955; Wilson and Osbourn, 1960; Mersmann et al., 1987; Ryan, 1990), birds (Wilson and Osbourn, 1960), reptiles (Bjorndal et al., 2003; Radder et al., 2007), and amphibians (Alford and Harris, 1988; Vonesh and Bolker, 2005), but most extensively in fish (Ali et al., 2003 for review). Despite the diversity of animals and plants that can exhibit CG, the underlying mechanisms governing the response are still poorly understood.

A broad range of teleosts are capable of undergoing CG responses following alleviation of various growth-stunting conditions or their combination, including suboptimal temperature, crowding, or other stressful environments, and feed restriction, the latter reflecting the condition most often studied (Ali et al., 2003). A CG response has been reported in salmonids (Dobson and Holmes, 1984; Jobling et al., 1993; Maclean and Metcalfe, 2001; Nikki et al., 2004), cyprinids (Russell and Wootton, 1992; Wieser et al., 1992), perciformes (Hayward et al., 1997; Picha et al., 2006, 2008b; Turano et al., 2007, 2008; Ferrando et al., 2009), flatfish (Cho, 2005; Heide et al., 2006), sticklebacks (Zhu et al., 2003), cichlids (Wang et al., 2000), catfish (Gaylord and Gatlin, 2000), and gadids (Jobling et al., 1994). While the degree of growth compensation achieved depends on species, it is nonetheless typically characterized by hyperphagia, improved feed conversion efficiency, and elevated specific growth rate (SGR). Although not often assessed, a critical feature of individuals undergoing CG is that their SGR is higher relative to similar-sized cohorts (i.e., SGR normalized to body mass) that were never subjected to stunting conditions (Skalski et al., 2005; Picha et al., 2006). Considering that CG results in enhanced growth rate and feed efficiency, it is not surprising that commercial production appears to be the driving impetus behind investigations into CG in fish, as the majority of studies to date involve cultivated species. Compared to conventional methods of fish farming that deploy a constant regimen, incorporation of rearing protocols that induce CG shows promise of reducing the amount of feed needed to grow at least some species of fish commercially.

**Figure 2:**
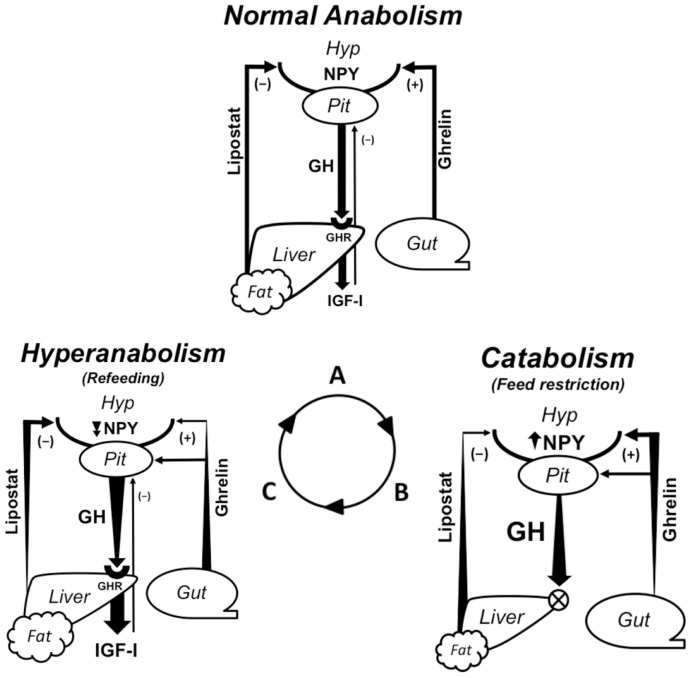
**Endocrine regulation of growth and appetite during normal anabolism, catabolism, and hyperanabolism (CG) resulting from feeding status**. Growth is regulated by the GH/IGF axis; GH secreted into circulation by the pituitary binds its receptor (GHR) to stimulate hepatic IGF-I production, which systemically drives somatic growth and exerts negative feedback on GH secretion. Lipolysis is an alternate function of GH during catabolism. Peripheral signals from a lipostatic mechanism (anorexigenic), possibly leptin, and ghrelin (orexigenic) regulate energy intake by modulating NPY and other neuropeptides in the central feeding center. Ghrelin also functions as a GH secretagogue. Arrows show the direction of regulatory pathways; widening/narrowing of arrows represents a dynamic increase/decrease in a component over the duration of a particular metabolic state. **(A)** During regular feeding, energy homeostasis is maintained by matching energy intake and expenditure. Peripheral signals counter-regulate appetite centrally. Growth is regulated by nominal levels of circulating GH, which stimulates IGF-I production via hepatic GHRs. **(B)** Fasting necessitates catabolic processes to provide energy for basal metabolism. Rising ghrelin production stimulates both appetite and circulating GH levels. Elevated lipolytic GH levels exploit stored energy reserves, decreasing lipostatic signaling. Reduced hepatic GHR expression desensitizes the liver to GH-induced IGF-I production. **(C)** Refeeding signifies the switch from catabolic to anabolic processes. Temporally elevated orexigens carried over from fasting drive hyperphagia. The return to positive energy status is characterized by the resumption of hepatic GH sensitivity and a steep rise in circulating IGF-I levels, which promotes accelerated growth. Eventually, the repletion of energy reserves and negative feedback from IGF-I returns GH and appetite to nominal levels, marking the return to normal growth rates. (PIT, pituitary; HYP, hypothalamus; NPY, neuropeptide Y; GH, growth hormone; GHR, growth hormone receptor; IGF-I, insulin-like growth factor I).

Because most fish exhibit indeterminate growth and many are susceptible to seasonal changes in growth rate associated with natural variations in temperature and prey availability, they tend to exhibit a robust capacity for CG (Mommsen, 2001). Hence, they can ostensibly serve as valuable subjects for evaluating the metabolic and endocrine mechanisms that may contribute to anabolic processes generally, and hyperanabolism specifically. Acknowledging the diversity of fish in which CG has been documented and the complexity of the response itself, few attempts have been made to consolidate what is known about the endocrine mechanisms that underlie the response; however, the cumulative research on isolated components of CG provides insightful information from which to extrapolate a fundamental framework. In particular, the CG response can be divided into catabolic (e.g., during fasting, stress, low temperature) and anabolic (during realimentation or a return to more favorable conditions) phases, which elicit distinct and sequential endocrine responses (Figure 2; Table 1). The purpose of this review is to ascribe possible roles to select appetite and growth-regulatory hormones in the context of the catabolic and (hyper)anabolic phases of the CG response in teleosts, with emphasis on growth hormone (GH), insulin-like growth factors (IGFs), cortisol, somatostatin, neuropeptide Y (NPY), ghrelin, and leptin.

**Table 1:** **Modulation of select endocrine factors during the transition from catabolism (fasting) to hyperanabolism (refeeding)**.

	Catabolism (*fasting*)			Hyperanabolism (*refeeding*)		
	Response	Effect	Reference	Response	Effect	Reference
**GH**	Elevated levels	Lipolysis (protein sparing)	Sheridan (1986), Deng et al. (2004), Albalat et al. (2005), Small and Peterson (2005), Norbeck et al. (2007), Picha et al. (2009)	Residually high, then decreasing	Elevated IGF production, enhanced protein uptake	Collie and Stevens (1985), Foster et al. (1991), Sun and Farmanfarmaian (1992), Fine et al. (1993), Norbeck et al. (2007), Picha et al. (2009), Pierce et al. (2011), Kling et al. (2012)
**GHR (liver)**	Downregulated	Hepatic GH resistance	Gray et al. (1992), Mori et al. (1992), Duan (1998), Deng et al. (2004), Saera-Vila et al. (2005), Norbeck et al. (2007), Picha et al. (2008b)	Upregulated	GH-induced IGF production	Gray et al. (1992), Small et al. (2006), Picha et al. (2008b)
**IGFs**	Suppressed	Growth stasis	Duan and Plisetskaya (1993), Picha et al. (2008b)	Elevated/Overcompensated	Enhanced somatic growth	Uchida et al. (2003), Beckman et al. (2004), Picha et al. (2008a); Picha et al. (2008b)
**Ghrelin**	Elevated levels	Increased appetite, GH secretion	Kaiya et al. (2003a); Ran et al. (2004), Unniappan and Peter (2004); Fox et al. (2007), Picha et al. (2009)	Residually high, then decreasing	Hyperphagia	Riley et al. (2005), Matsuda et al. (2006), Miura et al. (2006)
**NPY**	Elevated levels	Increased appetite	Peng et al. (1994), Silverstein et al. (1998), Leonard et al. (2001)	Residually high, then decreasing	Hyperphagia	Lopez-Patino et al. (1999), Narnaware et al. (2000), Volkoff and Peter (2001), Aldegunde and Mancebo (2006), Kiris et al. (2007)
**Leptin**	Species/tissue dependent	Regulation of energy metabolism?	Kling et al. (2009), Rønnestad et al. (2010), Fuentes et al. (2012), Frøiland et al. (2012), Trombley et al. (2012), Zhang et al. (2012)	Species/tissue dependent	Lipostatic signal?	Johnson et al. (2000), Nieminen et al. (2003), Volkoff et al. (2003), Murashita et al. (2008), Gorissen et al. (2009), Won et al. (2012)
**Cortisol**	Elevated levels	GH secretion, hepatic GH resistance, IGF-I suppression	Nishioka et al. (1985), Kajimura et al. (2003), Small and Peterson (2005), Leung et al. (2008), Pierce et al. (2011)	Low levels	Enhanced somatic growth	Kajimura et al. (2003), Leung et al. (2008)
**Somatostatin**	Elevated levels	Hepatic GH resistance, IGF-I suppression	Very and Sheridan (2002), Sheridan and Kittilson (2004)	Low levels	Enhanced somatic growth	Very and Sheridan (2002)

*The response, or relative presence of a component, during a particular metabolic state is paired with what is estimated to be the relevant effect it has in eliciting compensatory growth (GH, growth hormone; GHR, growth hormone receptor; IGFs, insulin-like growth factors; NPY, neuropeptide Y)*.

## Catabolic State: Priming the Compensatory Growth Response

In order to induce CG, a preceding catabolic period is necessary, the degree of which affects the overall magnitude of the response (Russell and Wootton, 1992; Wieser et al., 1992; Wang et al., 2000). This negative energy period depletes endogenous energy reserves and alters endocrine profiles that modulate appetite and growth potential once feeding is reinstated (Figure 2B). Brief periods of feed restriction do not sufficiently deplete stored energy or result in stunting, and can be countered with behavioral compensation such as decreasing energy expenditure (Ali et al., 2003). Excessively long periods of fasting, on the other hand, lead to an irrecoverable lapse in growth that prevents full catch up to fed cohorts (Bilton and Robins, 1973; Gaylord and Gatlin, 2000). Nevertheless, moderate catabolism that taps expendable energy-storing tissues physiologically primes the CG response by opening pathways that elevate circulating GH and stimulate orexigens such as ghrelin and NPY. Studies in striped bass (*Morone saxatilis*) suggest that a prerequisite drop in the hepatosomatic index (HSI) to about 1.5 is necessary in order to elicit the response (Picha et al., 2006; Turano et al., 2007). The rise in appetite and alterations in physiology that occur during the catabolic phase preceding CG thereby potentiate hyperphagia and accelerated growth when feeding is reestablished. Hence, an adequate, but not excessive level of catabolism is essential to elevating the capacity of an animal to undergo CG and possibly achieve full catch-up growth when conditions improve.

### Growth hormone: Function and regulation during catabolism

Under differential regulation by a host of neuroendocrine regulatory factors, GH serves dual roles depending on metabolic state, mobilizing lipids during catabolism and promoting somatic growth during anabolism (reviewed in Canosa et al., 2007). During fasting, rising plasma GH (MacKenzie et al., 1998), along with the related somatolactin in fish (Mingarro et al., 2002), protects non-expendable tissue such as muscle and vital organs from being catabolized by preferentially metabolizing fat over protein. This lipolytic function has been demonstrated in fish with exogenous GH treatment *in vivo* in coho salmon (*Oncorhynchus kisutch*; Sheridan, 1986) and *in vitro* in gilthead sea bream (*Sparus aurata*) adipocytes (Albalat et al., 2005), and is a critical adaptation to surviving negative energy periods.

Catabolically elevated GH secretion is mediated by reductions in metabolite levels (glucose/amino acids) and is stimulated *in vitro* and *in vivo* by orexigens, including ghrelin (Picha et al., 2009) and NPY (Peng et al., 1993), as well as by the lack of negative feedback inhibition from IGF-I. In striped bass pituitaries, IGF-I potently suppresses *in vitro* GH synthesis and release (Fruchtman et al., 2000; Picha et al., 2009), which likely contributes to elevated GH production during fasting when IGF-I levels are depressed. Cortisol, the dominant stress corticosteroid in fish (Mommsen et al., 1999; Dyer et al., 2004), also stimulates *in vivo* GH transcription in channel catfish (*Ictalurus punctatus*; Small and Peterson, 2005) and *in vitro* release from somatotrophs in Mozambique tilapia (*Oreochromis mossambicus*; Nishioka et al., 1985). Plasma GH levels are elevated by as much as twofold in rainbow trout (*Oncorhynchus mykiss*; Sumpter et al., 1991; Farbridge and Leatherland, 1992; Norbeck et al., 2007), Nile tilapia (*Oreochromis niloticus*; Toguyeni et al., 1996), channel catfish (Small and Peterson, 2005), and striped bass (Turano, 2007) during fasting when exogenous metabolic substrates are limited and fat reserves are needed for energy. Elevated GH is therefore able to mobilize lipids for maintenance of basal metabolism during food deprivation, but without directing limited energy resources toward growth due to catabolic GH resistance in the liver.

The actions of GH are mediated by GH receptors (GHRs), for which two distinct gene lineages exist in fish and which operate via different signaling pathways (Kittilson et al., 2011). The teleost GHR-1 is homologous to the single mammalian GHR (Fukamachi and Meyer, 2007) and has a shared affinity for both GH and somatolactin (Fukada et al., 2005) whereas the type 2 GHR is specific to GH activation (Fukada et al., 2004; Jiao et al., 2006; Pierce et al., 2007). The degree to which the individual receptor types mediate lipolysis or regulate growth is not known, but GH actions in various tissues are likely contingent on the differential expression of these receptors depending on whether catabolic or anabolic processes are required (de Celis et al., 2003; Saera-Vila et al., 2005; Very et al., 2005; Kittilson et al., 2011). During negative energy states, hepatic resistance to elevated plasma GH is evident as decreased IGF-I production (Duan and Plisetskaya, 1993; Picha et al., 2008b) due to catabolic suppression of ligand binding to GHRs (Gray et al., 1992; Mori et al., 1992; Duan, 1998). This type of hepatic GH resistance during catabolism is characterized as downregulated GHR mRNA levels in striped bass (Picha et al., 2008b) and gilthead seabream (Saera-Vila et al., 2005), decreased hepatic GH binding in gilthead sea bream (Pérez-Sánchez et al., 1994) and both reduced hepatic GHR transcript and GH-binding in rainbow trout (Norbeck et al., 2007) and black seabream (*Acanthopagrus schlegeli*; Deng et al., 2004); alleviated in all cases by refeeding.

Hepatic GH resistance is likely mediated, in part, by cortisol and somatostatins during fasting. While stimulating GH synthesis, cortisol simultaneously suppresses hepatic IGF-I production through direct downregulation of transcription and synthesis in silver sea bream (*Sparus sarba*; Leung et al., 2008) and Mozambique tilapia (Kajimura et al., 2003; Pierce et al., 2011), or in conjunction with suppression of GHR transcript in channel catfish (Small et al., 2006). Somatostatins also reduce GH binding in the liver and suppress IGF-I gene expression (Very and Sheridan, 2002; Sheridan and Kittilson, 2004). Hepatic resistance to GH through reduced receptor expression and signaling, despite elevated levels of circulating ligand, signifies an uncoupling of the lipolytic and growth-regulatory functions of GH during negative energy states. The carry over of catabolically elevated GH, in turn, helps drive enhanced IGF-I production and may ultimately potentiate hyperanabolic growth pending the return to a positive energy state when hepatic GHR signaling resumes.

### Ghrelin: Peripheral modulator of GH secretion and appetite during catabolism

The rise in lipolytic GH during fasting is fundamentally imperative for sparing muscle and organs by deferring catabolism to fat stores. Upstream regulation of enhanced GH production during fasting is therefore dependent on an indicator of nutritional state to coordinate catabolic lipolysis with negative energy states. The orexigenic peptide, ghrelin, responds to fasting and is a potent GH secretogogue, comparable in effect to growth hormone releasing hormone (GHRH) (Hataya et al., 2001). The differential regulation of appetite and GH secretion by ghrelin in fish relies on specific Ser3 modifications (Unniappan et al., 2002; Riley et al., 2005; Jönsson et al., 2007). Ghrelin’s actions are mediated by the growth hormone secretogogue receptor (GHSR), which is distinct from the GHRH receptor (Kojima et al., 1999). In fish, ghrelin mRNA is expressed predominantly in the gut and stomach (Unniappan and Peter, 2005), and it is interestingly the only known peripheral orexigen originating from the gut considering its proximity to nutrient uptake. To a lesser extent, central ghrelin gene expression has also been detected in rainbow trout (Kaiya et al., 2003a), eel (*Anguilla japonica*; Kaiya et al., 2003b), Mozambique tilapia (Kaiya et al., 2003c), and goldfish (*Carassius auratus*; Unniappan et al., 2002), as has GHSR transcript in black sea bream (Chan and Cheng, 2004). Ghrelin stimulates GH secretion *in vitro* in cultured orange spotted grouper (*Epinephelus coioides*) pituitaries (Ran et al., 2004) and *in vivo* and *in vitro* in Mozambique tilapia (Fox et al., 2007), rainbow trout (Kaiya et al., 2003a), goldfish (Unniappan and Peter, 2004), and striped bass (Picha et al., 2009). Besides actively stimulating GH secretion, in mammals ghrelin also independently acts as a functional antagonist to somatostatin (Arvat et al., 2001; Tannenbaum and Bowers, 2001; Tannenbaum et al., 2003), itself an inhibitor of GH secretion. Ghrelin is therefore capable of peripherally stimulating GH secretion through vagal afferents originating near the stomach, as well as by acting directly on the pituitary or through modulation of central GH release factors.

Ghrelin is also a potent appetite stimulant, serving as a peripheral signal to the brain during periods of negative energy balance (Banks et al., 2002; Cummings et al., 2004). Orexigenic ghrelin signaling operates via vagal afferents as well as central pathways paralleling those of GH regulation, although appetite and GH stimulatory pathways are independent (Tschop et al., 2000; Wren et al., 2000; Nakazato et al., 2001; Date et al., 2002). The orexigenic properties of ghrelin have been reviewed in mammals (Anderson et al., 2005; Ueno et al., 2005) and fish (Unniappan and Peter, 2005; Kaiya et al., 2008). Appetite was stimulated by a single IP/ICV injection of homologous ghrelin in goldfish (Matsuda et al., 2006; Miura et al., 2006) and by chronic treatment in tilapia (Riley et al., 2005). The effect of ghrelin on appetite is less clear in salmonids, which undergo seasonal alterations in feeding behavior (Metcalfe et al., 1986; Metcalfe and Thorpe, 1992). In juvenile rainbow trout, IP injection of rat ghrelin was orexigenic (Shepherd et al., 2007) as in other fish, although both IP and ICV delivery of native ghrelin suppressed feeding (Jönsson et al., 2010) in similar-sized trout or had no effect (Jönsson et al., 2007) when injected IP in larger fish. These variable results suggest that ghrelin may have different functions depending on life stage in salmonids, and that homologous and heterologous peptides could account for the different effects observed in these studies. Future research will need to consider these contingencies.

In accord with its orexigenic function, ghrelin levels tend to rise during fasting and decline after feeding in mammals (Ueno et al., 2005) as well as in fish (Unniappan et al., 2004; Canosa et al., 2005; Matsuda et al., 2006; Terova et al., 2008; Picha et al., 2009). Exceptions were observed in tilapia (Riley et al., 2008) and rainbow trout (Jönsson et al., 2007), however, and may represent species-specific differences in regulation of the ghrelin system among teleosts. Ghrelin nonetheless appears to be an important peripheral signal for regulating both GH secretion and appetite based on nutritional status. Studies further suggest that stimulation of ghrelin production during fasting and the lag time between refeeding and the return of ghrelin to basal levels may depend on the degree to which catabolic processes deplete energy reserves. In the short term, gut and hypothalamic preproghrelin mRNA along with circulating ghrelin levels increased on the order of days in fasted goldfish, then went down within several hours after refeeding (Unniappan et al., 2004). European seabass (*Dicentrarchus labrax*) stomach ghrelin mRNA levels increased over 35 days of fasting, then dropped back to basal levels after 10 days of refeeding (Terova et al., 2008). Striped bass exhibited elevated plasma ghrelin levels after 3 weeks of fasting and a 43-fold increase relative to fed controls after continued fasting and cold-banking for 90 days, followed by a return to baseline within 21 days of refeeding and temperature warm up (Picha et al., 2009). Plasma ghrelin and GH levels in the treatment fish of this trial were concordantly elevated during fasting and then eventually returned to control levels after refeeding. Refeeding was additionally marked by hyperphagia and full catch up growth. Picha et al. (2009) show that *in vitro* stimulation of GH by ghrelin occurs in somatotrophs derived from both continually fed and fasted fish, but not from refed fish, suggesting the hypophyseal GHSR may be downregulated during CG, which would contribute to the eventual decline in GH seen during refeeding of fasted fish. Taken together, the trends in ghrelin regulation and function observed in fish suggest that catabolically elevated ghrelin simultaneously raises lipolytic plasma GH while priming a hyperphagic response during refeeding. The coincidence of these effects likely contributes significantly to the CG response, and may in part explain why a catabolic phase is needed to precede hyperanabolism.

### Central modulators of appetite

Appetite is increased during fasting through the upregulation of central orexigenic neuropeptides (Kalra et al., 1999), with similar hormones existing in mammals and fish. In the latter, a number of orexigens are expressed in the pre-optic hypothalamic region (Volkoff et al., 2005), which appears to be the teleost analog to the mammalian feeding center. Central injection of NPY (Lopez-Patino et al., 1999; Narnaware et al., 2000; Aldegunde and Mancebo, 2006; Kiris et al., 2007), galanin (de Pedro et al., 1995; Volkoff and Peter, 2001), and orexins (Volkoff et al., 1999) stimulate appetite in teleosts. While NPY is considered the most potent orexigen in fish and has garnered the most research, these other central peptides interact with NPY to augment appetite in response to negative energy status (Volkoff et al., 2005).

Central NPY (Peng et al., 1994; Silverstein et al., 1998; Leonard et al., 2001) and AgRP (Cerdá-Reverter and Peter, 2003) mRNA is regulated by nutritional state and increases during negative energy states to promote energy intake. Ghrelin stimulates central gene expression of NPY, which has been shown to mediate the orexigenic effects of ghrelin in goldfish (Miura et al., 2006). As in mammals (Stephens et al., 1995; Schwartz et al., 2000), the anorexigen, leptin, suppresses the effects of exogenous NPY on appetite in fish (Lin et al., 2000; Volkoff et al., 2003). In rodents, appetite is stimulated by NPY when ghrelin levels are high and leptin low (Bagnasco et al., 2002), or during periods of negative energy balance; however, leptin’s regulation by metabolic state in fish is equivocal (discussed below). The upregulation and interaction of central orexigenic neuropeptides during fasting likely culminates in the hyperphagic response during refeeding, thus providing the substrate and energy necessary for a CG response to occur.

## Refeeding: Hyperphagia and Hyperanabolism (Compensatory Growth)

Hyperanabolism, or the accelerated growth phase that characterizes CG, is the result of hyperphagia and heightened growth axis activity during refeeding, particularly in the rapid rise in IGF-I production that occurs when hepatic sensitivity to GH returns. As discussed in the first half of this review, CG is preceded by a catabolic phase that primes an organism for hyperanabolism. If the endurance of the catabolic state is sufficient, and food is ample when feeding resumes, then a temporal hyperphagic response is elicited and a net positive energy state achieved through the reintroduction of exogenous energy and metabolic substrates. When energy stores are regained, orexigenic signaling declines and hyperphagia subsides. Under these terms, an organism exhibits lipostatic regulation of energy homeostasis (Jobling and Johansen, 1999), a system in which energy reserves are maintained within a certain range by endocrine signals derived from energy-storing tissues that regulate feeding and energy expenditure. CG seems to occur during the lag time between refeeding and the lipostatic abatement of hyperphagia and enhanced growth axis activity.

### Hyperphagia and assimilation efficiency

Hyperphagia is an integral component of CG (Ali et al., 2003) and is a common response to energy deficit in a variety of fish; European minnow (*Phoxinus phoxinus*; Russell and Wootton, 1992), Atlantic salmon (*Salmo salar*; Bull and Metcalfe, 1997), centrarchid sunfish (genus *Lepomis*; Hayward et al., 1997), Nile tilapia (Wang et al., 2000), striped bass (Picha et al., 2008b; Turano et al., 2008), and stickleback (*Gasterosteus aculeatus*; Zhu et al., 2003). The magnitude of the hyperphagic response depends on the duration of fasting in salmon, and appears to be largely influenced by the degree to which lipid reserves are depleted by catabolic processes (Bull et al., 1996). Hyperphagia during CG is attributable to catabolically elevated levels of orexigens that are upregulated during negative energy states.

Hyperanabolism during refeeding is fueled by an influx of metabolic substrates that are rapidly allocated to somatic growth through heightened mitogenic activity of the growth axis; however, hyperphagia alone may not account for the accelerated growth rate experienced during CG. Gurney et al. (2003) propose, through energetics modeling, that high substrate assimilation rates during hyperphagia drive CG by partitioning resources specifically to skeletal growth rather than to energy reserve deposition. Skalski et al. (2005) elaborate on the energetics model of hyperanabolism, suggesting that physiological changes, including increased assimilation efficiency during feeding and reduced mass-specific maintenance costs during fasting, work in conjunction with hyperphagia to drive CG in striped bass. A subsequent study that normalizes SGR to body size supports that the growth rate is significantly higher in fasted/refed hybrid striped bass relative to controls (Picha et al., 2008b), and is not merely an allometric artifact of smaller, stunted fish compared to larger, fed cohorts.

The mechanism that adjusts energy allocation around assimilation rate is undefined, although the lipolytic and growth promoting functions of GH, along with its regulatory profile under variable metabolic conditions, suggests an influential role in optimizing substrate conversion to skeletal growth. Long-term GH treatment in rainbow trout improved feed conversion by 60% (Kling et al., 2012). Exogenous GH treatment enhanced amino acid uptake (Collie and Stevens, 1985) as well as growth rate, appetite, and food conversion in coho salmon (Markert et al., 1977). In fed striped bass, weekly bovine GH injection increased the number of intestinal amino acid transporters and intestinal mass (Sun and Farmanfarmaian, 1992), suggesting improved protein uptake capacity. Similarly in carp (*Cyprinus carpio*; Fine et al., 1993) and rainbow trout (Foster et al., 1991), protein assimilation, and feed conversion were improved by chronic GH treatment. The carry over of catabolically elevated GH after refeeding, in addition to driving somatic growth through stimulation of IGFs, may also improve protein assimilation at a time when substrates are in abundance due to elevated feeding (Peter and Marchant, 1995; MacKenzie et al., 1998).

### Hyperanabolism: Augmentation of the GH/IGF growth axis

Compensatory growth ultimately refers to the rapid growth, or hyperanabolic, response that occurs during the feeding of previously fasted animals, and which allows them to recover lost growth opportunity. During positive energy states, circulating GH binds hepatic GHRs to induce production and secretion of IGF-I, the prominent mitogen responsible for somatic growth in vertebrates (Froesch et al., 1985; Picha et al., 2008a). Like IGF-I, hepatic IGF-II transcription is also stimulated by GH in a broad range of fish (Shamblott et al., 1995; Duguay et al., 1996; Carnevali et al., 2005; Gabillard et al., 2006; Moriyama et al., 2008a,b; Pierce et al., 2011) and remains responsive to GH into adulthood. Hepatic GHR transcription positively correlates with GH binding in gilthead sea bream (Pérez-Sánchez et al., 1994) and with circulating IGF-I levels in channel catfish (Small et al., 2006) during fasting and refeeding, corroborating that metabolic state mediates GH signaling through changes in GHR expression in the liver where the majority of endocrine IGFs are produced (Reinecke and Collet, 1998; Vong et al., 2003; Terova et al., 2007). Chronic GH treatment increases growth rate in a diverse range of fish (Markert et al., 1977; Weatherley and Gill, 1987; Agellon et al., 1988; Cavari et al., 1993), suggesting that the catabolically elevated GH levels that temporarily persist during refeeding may contribute to an accelerated growth rate by stimulating the growth axis. The return of hepatic sensitivity to GH during refeeding, which reinstates IGF synthesis, is therefore critical to CG. The duration of high plasma GH levels may depend on catabolic history and the extent to which energy reserves are depleted during fasting, but appears to decline back to normal levels within 2 weeks of refeeding in rainbow trout and striped bass (Norbeck et al., 2007; Picha et al., 2009).

Consistent with the mitogenic attributes of IGFs, plasma IGF-I (Uchida et al., 2003; Beckman et al., 2004) and hepatic IGF-I and II mRNA (Picha et al., 2008b) levels positively correlate with SGR in fasted and refed fish. The relative change in circulating IGF-I over a growth increment is an even better corollary to SGR in striped bass than absolute IGF-I levels (Picha et al., 2006), which may indicate enhancement of IGF receptor sensitivity during the transition from catabolic back into anabolic states as plasma IGF-I levels are in the process of rising. The steep rebound of depressed plasma IGF-I levels during the refeeding of fasted striped bass corresponded directly to the hyperanabolic phase of the growth curve (Picha et al., 2008b). Moreover, transcript levels of hepatic GHRs and IGFs in these fish during refeeding actually exceeded those of constantly fed control fish, suggesting that overcompensation in expression of key growth-regulatory hormones may be contributing significantly to the accelerated growth that occurs with CG (Picha et al., 2008b). This overcompensation is further exacerbated in striped bass when the alleviation of a previous period of feed restriction is combined with cold-banking, similar to what wild fish experience during spring warm up when temperature and prey availability are more optimal for growth (Perez-Sanchez and Le Bail, 1999; Mingarro et al., 2002; Picha et al., 2009). Similarly, overcompensation in circulating IGF-I concomitant with enhanced activation of IGF-I signaling in muscle tissue was observed in fine flounder (*Paralichthys adspersus*; Fuentes et al., 2011). An elevation in muscle IGF-I mRNA levels has been observed in a number of other fish (Chauvigne, 2003; Montserrat et al., 2007a,b; Picha et al., 2008b; Kling et al., 2012), suggesting a parallel autocrine or paracrine mechanism within the skeletal tissue itself. Taken together, the coordinated dynamics of the GH/IGF growth axis appears key in eliciting CG, whereby the expression levels of and sensitivity to growth-regulatory hormones is increased relative to normal animals on a continuous regimen, subsequently resulting in a hyperanabolic state characteristic of CG.

### The lipostatic return to energy homeostasis

The CG response to fasting is finite, attenuating once lost energy resources re-accumulate and hyperphagia abates. The lipostatic model of energy homeostasis proposes that adiposity acts as a regulatory mechanism on appetite in order to maintain a threshold of energy deposition (Kennedy, 1953). Kennedy observed that depletion of adipose stores in fasted rats stimulated feeding, which returned to normal levels when reserves returned to a critical mass. Studies support the presence of a teleost lipostat-like mechanism, as well, although lipid partitioning can vary considerably between adipose tissue, liver, and muscle in different fishes (Dias et al., 1999; Frøiland et al., 2012). The hyperphagic response to long-term fasting in salmonids appears to be driven by a decrease in whole body lipid content and terminates, along with the CG response, when proximate composition is restored (Jobling and Miglavs, 1993; Johansen et al., 2001). Fluctuating gross lipid levels during fasting and refeeding similarly suggest lipostatic regulation of hyperphagia in three-spined stickleback (Zhu et al., 2003) and striped bass (Turano et al., 2007) undergoing CG. The liver is a significant lipid storing and metabolizing tissue in some fish, and may also be involved in sensing and maintaining energy reserve levels. More so than visceral adipose mass, changes in the HSI during cycles of feed deprivation and refeeding in striped bass, in which liver is a major lipid storage organ, are indicative of metabolic state and the likelihood of achieving an elevated SGR, and hence CG, during refeeding (Picha et al., 2006; Turano et al., 2008).

### Is leptin the teleost lipostatic hormone?

The mechanism that represents overall energetic status, endocrine or otherwise, is not well defined in teleosts, in part because different fish partition stored energy in various locations. In mammals, the anorexigenic peptide hormone, leptin, reflects fat deposition. Leptin modulates food consumption and energy expenditure according to endogenous energy availability, and is considered the primary lipostatic hormone (Ahima and Flier, 2000; Arora and Arora, 2008). If leptin functions as a lipostat in teleosts as well, it would likewise need to be indicative of energy availability. Even though leptin is consistently anorexigenic in fish as in mammals, evolutionarily isolated gene duplication events, physiological differences in energy storage and diverse life histories may underlie divergent functions for leptin in fish that only partially resemble those of higher vertebrates.

Leptin centrally regulates feeding by stimulating appetite-suppressing neuropeptides and inhibiting appetite-stimulating neuropeptides in mammals (Ahima et al., 1999; Elias et al., 1999; Zhang and Felder, 2004) and fish (Volkoff et al., 2003; Murashita et al., 2008). Leptin injection accordingly reduces feeding in goldfish (de Pedro et al., 2006), rainbow trout (Murashita et al., 2008), and striped bass (Won et al., 2012). While its anorexigenic property logically integrates into a system in which leptin serves as a lipostat, such as the mammalian paradigm, it fits less aptly into a system where leptin expression may not correlate with energy reserves, as is the case for some teleosts.

Studies evaluating leptin responsiveness to metabolic state in teleosts are equivocal. Circulating leptin (Johnson et al., 2000; Nieminen et al., 2003) or mRNA levels in liver (Gorissen et al., 2009; Won et al., 2012) are depressed in some fish during fasting, or otherwise reflect energy deposition (Kling et al., 2012). Conversely, some species exhibit rising plasma (Kling et al., 2009; Fuentes et al., 2012; Trombley et al., 2012) or gene expression levels in lipid storing tissues (Rønnestad et al., 2010; Frøiland et al., 2012; Gambardella et al., 2012; Trombley et al., 2012; Zhang et al., 2012) during feed restriction, while others show no long-term regulation by feeding regimen (Huising et al., 2006). Leptin gene expression profiles in different energy-storing tissues during altered metabolic states vary, even among closely related species, and are not necessarily concomitant with plasma levels. These studies call into question whether leptin functions as a lipostatic endocrine signal aimed at mobilizing surplus energy stores or might instead drive other catabolic processes in fish. It is important to consider that some fish possess multiple leptin genes (Huising et al., 2006; Kurokawa and Murashita, 2009; Rønnestad et al., 2010; Zhang et al., 2012) arising from different genome duplication events within lineages, and that these paralogs may have different roles. If a lipostatic function for leptin, or one form of leptin, is inherent in fish, then the replenishment of energy reserves during refeeding may eventually attenuate hyperphagia and mark the end of the CG response through leptin signaling. If not, then further studies will be needed to reconcile the paradox of rising plasma leptin levels during fasting in some fish in light of its conserved anorexigenic property.

### Summary: Sequence of events during catabolic and anabolic states leading to CG

Compensatory growth is a period of accelerated growth following the alleviation of growth-stunting conditions, such as fasting, that potentially allows an organism to make up for lost growth opportunity (Figure 1). The response can be divided into a catabolic phase, when growth is impeded and energy reserves are tapped, and a hyperanabolic phase, when growth resumes at an elevated rate. This review chronologically describes physiological adjustments and endocrine activity in fish during these metabolic phases, and suggests how they may ultimately make CG possible through enhanced feeding, substrate assimilation, and rapid growth.

During the precedent catabolic phase (Figure 2B), orexigens stimulate appetite as endogenous energy reserves are depleted. Ghrelin and cortisol stimulate GH production and elevate circulating GH levels in order to free energy-storing lipids. However, hepatic GH resistance and the growth-inhibitory effects of cortisol and somatostatins suppress IGF production under the pretext that conditions are unfavorable for growth. Reduced negative feedback from IGF-I further permits GH levels to rise. A critical period of catabolism is therefore required to induce hormonal changes that prime the fish for hyperphagia and super-potentiate the growth axis.

During refeeding (Figure 2C), CG is fueled by the hyperphagic influx of exogenous energy and substrates. Food is assimilated with heightened efficiency as the result of modifications to metabolic substrate absorption, in part attributable to residually elevated GH levels. Hepatic GHRs are reinstated and GH sensitivity returns, followed by a steep rise or even overcompensation in IGF-I production. Production of IGFs during refeeding is also influenced by a decline in growth-inhibitors. Substrate and energy availability, enhanced assimilation efficiency, and augmentation of the growth axis culminate in a hyperanabolic, rapid growth phase until a lipostat-like mechanism initiates the return to basal appetite and a growth axis profile representative of a normal growth trajectory (Figure 2A).

Compensatory growth is therefore regulated by sequential endocrine responses during distinct metabolic states (Table 1). Teleosts have been the subject of numerous CG studies; however, our understanding of their endocrine mechanisms during this phenomenon is commonly limited to observations in a few model species or extrapolations from studies in higher vertebrates. While by no means inclusive, this theoretical composite of documented hormonal activity during catabolic and anabolic states is intended to provide a basic framework of the endocrine regulation of CG in fish, and perhaps higher vertebrates. The relevance of certain hormones, time frames, and even the potential of the CG response itself are likely contingent on species, size, and life stage. Nonetheless, the variables presented in this review are estimated to be of fundamental importance to CG in fish, although the degree of their relevance in particular species may vary.

## Conflict of Interest Statement

The authors declare that the research was conducted in the absence of any commercial or financial relationships that could be construed as a potential conflict of interest.
